# AGE-RELATED DIFFERENCES IN DAILY EXPERIENCES OF HAPPINESS: THE ROLE OF THINKING ABOUT THE FUTURE

**DOI:** 10.1093/geroni/igad104.1821

**Published:** 2023-12-21

**Authors:** Yoonseok Choi, Jennifer Lay, Minjie Lu, Da Jiang, Helene Fung, Peter Graf, Christiane Hoppmann

**Affiliations:** University of British Columbia, Vancouver, British Columbia, Canada; University of Exeter, Exeter, England, United Kingdom; Beijing Normal University at Zhuhai, Zhuhai, Guangdong, China (People’s Republic); Education University of Hong Kong, Hong Kong, Hong Kong; The Chinese University of Hong Kong, Hong Kong, Hong Kong; University of British Columbia, Vancouver, British Columbia, Canada; University of British Columbia, Vancouver, British Columbia, Canada

## Abstract

We examined the role of future time perspective (thinking about the future) in shaping age-related differences in time-varying experiences of happiness. Older adults’ experience of happiness is more strongly associated with low-arousal than with high-arousal positive affect. Low-arousal positive affective states may be conducive to engaging in meaningful social interactions with close others (e.g., listening, adjusting to others) and therefore serve key socio-emotional goals that are prioritized when future time is perceived as limited. We hypothesized that thinking less about the future would be related to stronger associations between happiness and low-arousal positive states in older than younger adults. We used daily life assessments from 258 participants (M = 48.4 years; 68% female; 77% Asian; 73% post-secondary education), which comprised older (M = 63.4 years) and younger (M = 20.1 years) adult samples collected at two locations (Vancouver, Canada; Hong Kong). Participants reported on their momentary affective states and thinking about the future (0-100 scales) up to 30 times over 10 days. Results replicate previous findings by showing that momentary happiness was more strongly associated with momentary calmness and more weakly associated with momentary excitement among older as compared to younger adults. Younger adults reported thinking more about the future than older adults. Thinking less about the future was related to stronger happiness-calmness and weaker happiness-excitement associations in daily life for older participants, only; for younger participants, it was associated with weaker happiness-calmness associations. Age and future thinking-related contours of happiness are discussed in the context of emotional aging theories.

